# Neural Cell Responses Upon Exposure to Human Endogenous Retroviruses

**DOI:** 10.3389/fgene.2019.00655

**Published:** 2019-07-11

**Authors:** Joel Gruchot, David Kremer, Patrick Küry

**Affiliations:** Department of Neurology, Neuroregeneration, Medical Faculty, Heinrich-Heine-University, Düsseldorf, Germany

**Keywords:** human endogenous retrovirus, neurodegenerative diseases, neurons, glia, mobile genetic elements

## Abstract

Human endogenous retroviruses (HERVs) are ancient retroviral elements, which invaded the human germ line several million years ago. Subsequent retrotransposition events amplified these sequences, resulting in approximately 8% of the human genome being composed of HERV sequences today. These genetic elements, normally dormant within human genomes, can be (re)-activated by environmental factors such as infections with other viruses, leading to the expression of viral proteins and, in some instances, even to viral particle production. Several studies have shown that the expression of these retroviral elements correlates with the onset and progression of neurological diseases such as multiple sclerosis (MS) and amyotrophic lateral sclerosis (ALS). Further studies provided evidence on additional roles for HERVs in schizophrenia (SCZ). Since these diseases are still not well understood, HERVs might constitute a new category of pathogenic components that could significantly change our understanding of these pathologies. Moreover, knowledge about their mode of action might also help to develop novel and more powerful approaches for the treatment of these complex diseases. Therefore, the main scope of this review is a description of the current knowledge on the involvement of HERV-W and HERV-K in neurological disease specifically focusing on the effects they exert on neural cells of the central nervous system.

## Involvement of HERVs in Neurological Diseases

Up to 8% of the human genome are of retroviral origin. These retroviral elements, termed human endogenous retroviruses (HERVs), invaded the germ line millions of years ago and have been permanently integrated into the genome of our primate ancestors (Küry et al., 2018). Following integration, retrotransposonal activity led to the amplification of these retroviral elements (Belshaw et al., 2004). While most of these retroviral genes contain intragenic deletions or nonsense mutations and are therefore presumed to be silent, some of them retained parts of their functionality and developed into enhancers of the immune defense (Grandi and Tramontano, 2018). Other genes, such as syncytin encoded by ERVWE1, a full length provirus at locus 7q21.2 on chromosome 7, were domesticated and act in placental development (Mi et al., 2000). HERV elements may normally be expressed at low levels, but environmental factors, such as hypoxia (Brutting et al., 2018), drugs (Liu et al., 2013), other viruses (Liu et al., 2017), and certain mutations (Yu et al., 2014), were shown to increase their expression. Importantly, several studies were able to show that inflammation plays a major role in HERV activation (Mameli et al., 2007; Mameli et al., 2013; Li et al., 2015; Manghera et al., 2015; Manghera et al., 2016b;Hurst and Magiorkinis, 2017). However, current research indicates that HERVs are also associated with several neurological disorders such as multiple sclerosis (MS; Perron et al., 1989), amyotrophic lateral sclerosis (ALS; Mccormick et al., 2008), and schizophrenia (SCZ; Yolken et al., 2000), which warrants research into underlying mechanisms of activation as well as into their role in disease etiology. As underlying disease causes of many neurological conditions remain elusive, HERV-directed research might shed light on new interactions and pathological processes implicated into disease onset or progression. These entities, which are *sensu stricto* neither viruses nor physiological genes, must therefore be considered as a new category of pathogenic elements (Feschotte and Gilbert, 2012). In the present review, we will summarize what is currently known about the involvement of HERVs in neurological diseases and will specifically address functions exerted on neurons and glial cells of the central nervous system (CNS).

Of note, HERV-W has been associated, repeatedly and based on a larger number of independent studies, with MS as recently reviewed in (Dolei, 2018). This demyelinating CNS disease of unknown etiology features miscellaneous clinical symptoms such as sensory, motor, and cognitive dysfunctions. Pathophysiologically, MS is characterized by immune cell infiltration, focal inflammation, and loss of myelin sheaths, leading to white and gray matter lesions and brain atrophy (Reich et al., 2018). Axonal degeneration, observed mainly but not exclusively during progression and later disease stages (Trapp et al., 1998), is another of its hallmarks and results in irreversible deficits. Mechanistically, direct autoimmune attacks on neurons (Derfuss et al., 2010) as well as secondary effects in response to myelin loss are responsible for axonal impairment and loss. In 1989, an association between retroviral elements and MS was described based on the analysis of primary leptomeningeal cell cultures isolated from MS patients (Perron et al., 1989). While these isolated viral particles were initially termed multiple sclerosis associated retrovirus (MSRV), it was later found that MSRV belongs, in fact, to the HERV family (Dolei and Perron, 2009; Perron and Lang, 2010). Follow-up studies provided convincing evidence that activation and expression of otherwise dormant HERV-W DNA sequences and the subsequent production of the encoded envelope (ENV) protein can trigger an immune response (Perron et al., 2001; Rolland et al., 2005). Moreover, it was shown that HERV-W ENV RNA and protein levels are increased in the cerebrospinal fluid (CSF) and serum of MS patients but rarely in healthy individuals (Garson et al., 1998; Mameli et al., 2009; Perron et al., 2012). Furthermore, it was shown that HERV-W ENV activates the innate immunity, priming it against myelin proteins. Accordingly, HERV-W ENV can act as an adjuvant in a model of experimental autoimmune encephalitis (EAE), which, in turn, can be rescued by the application of an HERV-W ENV-targeted therapeutic IgG4 antibody termed GNbAC1 (EAE; Perron et al., 2013). MS histology then revealed that the HERV-W ENV protein is mainly expressed by myeloid cells (Kremer et al., 2013; Van Horssen et al., 2016). Of note, a similar correlation was observed for HERV-W and chronic inflammatory demyelinating polyneuropathy (CIDP), an inflammatory, demyelinating disease of the peripheral nervous system (PNS; Faucard et al., 2016).

Apart from roles in cancer (Grabski et al., 2019) HERV-K also appears to be involved in a subpopulation of patients with sporadic ALS. This neurodegenerative disease is characterized by the progressive loss of both cortical and spinal motor neurons (Mathis et al., 2017). Although first described in the 19th century, its pathogenesis is still poorly understood despite considerable efforts to identify causes and susceptibilities in recent decades. Elevated HERV-K reverse transcriptase (RT) activity was observed in both blood and CSF from ALS patients (Macgowan et al., 2007; Mccormick et al., 2008). So far, two loci could be identified in the 7q34 and 7q36.1 regions, leading to the expression of HERV-K elements in ALS patients (Frank et al., 2005). Initial analysis of brain autopsy tissue revealed the expression of several HERV-K transcripts in cortical and spinal neurons of ALS but not in healthy control individuals (Douville et al., 2011). Although evidence for such an involvement is increasing (Meyer et al., 2017), it is currently challenged by a recent independent study that was not able to confirm the association between elevated cortical HERV-K RNA levels and ALS (Garson et al., 2019). Despite these conflicting observations related to the detection in ALS, it must be emphasized that transgenic mice expressing the HERV-K envelope protein display progressive motor dysfunction and motor cortex volume loss (Li et al., 2015).

SCZ is a complex neuropsychiatric disorder characterized by a variety of cognitive, emotional, and perceptual disturbances. Pathophysiologically, SCZ features decreased brain volume, loss of myelin, and altered astrocyte function (Archer, 2010). In contrast to MS and ALS, both HERV-W and HERV-K have been weakly linked to SCZ based on PCR amplification from CSF and post-mortem brains as well as on protein antigenemia (Yolken et al., 2000; Karlsson et al., 2001; Frank et al., 2005; Perron et al., 2008), while another study revealed upregulation of HERV-W ENV transcripts in plasma samples of SCZ patients (Huang et al., 2011). Moreover, a new study provides evidence that, in early stages of this disease, HERV-K methylation in peripheral blood is reduced (Mak et al., 2019). Of note, these observations contradict an earlier report suggesting that HERV-W expression is reduced in SCZ patients (Weis et al., 2007). The disparity between these reports may reflect different experimental approaches or a differential use of anti-psychotic medications in SCZ patients.

## Mechanisms of HERV Activation

It is known that silenced HERVs can be specifically activated and expressed in several neurological conditions based on complex underlying activation mechanisms. In this regard, numerous studies have established links between HERV activation and infections with viruses such as the Epstein Barr virus (EBV). In this context, EBV glycoprotein350 (EBVgp350) was found to trigger expression of HERV-W ENV in blood cells and astrocytes, possibly contributing to the onset of MS (Mameli et al., 2012; Mameli et al., 2013). Likewise, EBV was also shown to trigger HERV-K expression (Sutkowski et al., 2001). Similar activation mechanisms were demonstrated for *Herpesviridae* HSV1 and HHV6 (Perron et al., 1993; Ruprecht et al., 2006; Brudek et al., 2007; Charvet et al., 2018), providing a possible underlying mechanism explaining the well-established epidemiological link between these viruses and the susceptibility for MS. In addition, a direct involvement of the human immunodeficiency virus (HIV) Tat protein in activating HERV-W ENV in peripheral blood mononuclear cells (PBMCs), monocyte/macrophages, and astrocytes was described (Uleri et al., 2014). Of note, in monocytes, HIV Tat inhibits the expression of syncytin-1, whereas in differentiated macrophages it is stimulated (Uleri et al., 2014). In this regard it is important to note that HERV-K plasma levels were found to positively and negatively correlate with HIV infection and antiretroviral therapy, respectively (Bowen et al., 2016). Whether HIV infection as such or antiretroviral treatment account for this observation is currently debated.

Yet, another important activator of HERV expression is the nuclear factor kappa-light-chain-enhancer of activated B-cells (NF-κB) signaling pathway, based on an earlier report demonstrating that the pro-inflammatory cytokine tumor necrosis factor (TNF) α can stimulate the, at that time so-called, ERVWE1/syncytin promoter *via* NF-κB (Mameli et al., 2007). Similarly, HERV-K expression was also shown to respond to TNFα/NF-κB signaling (Li et al., 2015; Manghera et al., 2015; Manghera et al., 2016b). Such signaling could be part of a regulatory feedback loop, taking into account that HERV long terminal repeat (LTR)-sequences act as promoters for pro-inflammatory cytokine genes (Hurst and Magiorkinis, 2017).

In human SH-SY5Y neuroblastoma cells, caffeine and aspirin were shown to induce HERV-W ENV and GAG (group-specific antigen) transcription, providing a possible link between environmental factors, drugs, and endogenous virus activation (Liu et al., 2013). Whether such exogenous triggers can also affect HERV-W induction in myeloid cells, which are highly relevant for MS (Kremer et al., 2013; Van Horssen et al., 2016), remains to be demonstrated. Regarding ALS, TAR DNA binding protein 43 (TDP43), which is involved in the sporadic form of the disease (Mackenzie and Rademakers, 2008), was found to bind to LTR sequences, leading to the expression and accumulation of HERV-K (Li et al., 2015; Manghera et al., 2016a). A further contribution to HERV-W activation in MS was proposed to be mediated *via* endoplasmic reticulum (ER) stress (Deslauriers et al., 2011). Finally, progerin, a nuclear protein involved in the accelerated aging Hutchinson–Gilford progeria syndrome, was found to strongly downregulate transcription of all classes of repetitive sequences including HERVs in dopaminergic neurons generated from induced pluripotent stem cells (Arancio, 2019). Whether corresponding lessons can be learned in light of neurodegeneration in MS or ALS needs to be shown in future. Of note, progerin was also shown to impair the nuclear factor erythroid 2–related factor 2 (Nrf2)-mediated anti-oxidative response (Kubben et al., 2016), a mechanism implicated in MS neuroprotection (Linker et al., 2011).

## HERV Effects Exerted on Neural Cells

### Neurons

**Figure 1:**
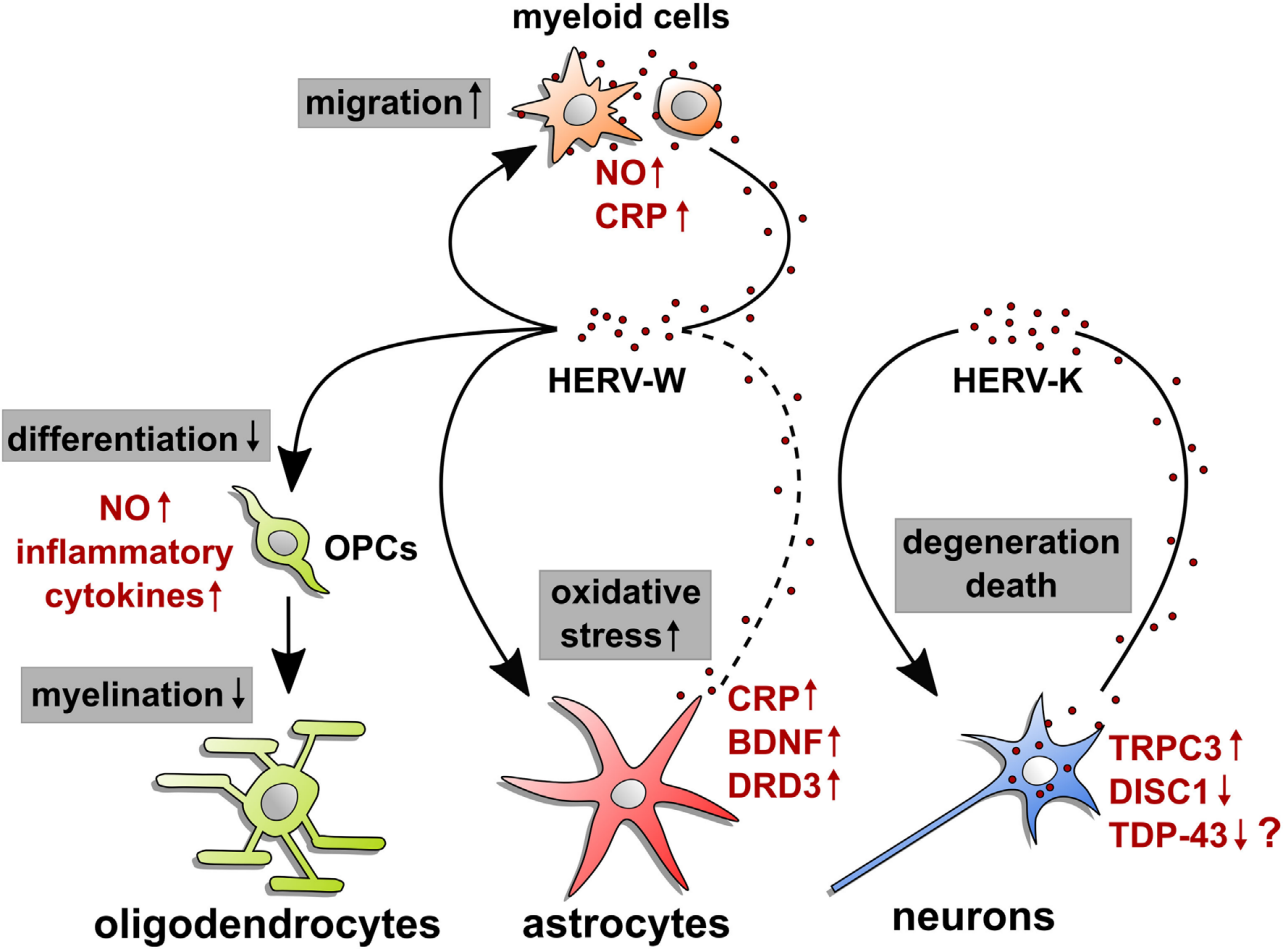
HERV-mediated effects on neural cells. This illustration summarizes origin and observed molecular effects of HERW-W and HERV-K on cells of the central nervous system. Arrow starting points indicate cellular sources of HERV particles or proteins (red dots), whereas arrowheads point to influenced cell types. Modulated processes are shown in gray boxes, and regulated molecules are highlighted in red next to each cell type. The question mark next to TDP-43 refers to its postulated regulation in neurons. Whether microglia and astroglia respond to HERVs in an auto- and/or paracrine way and whether neurons react to internal and/or extracellular HERVs remains to be shown. OPCs: oligodendroglial progenitor cells; NO: nitric oxide; CRP: C-reactive protein; BDNF: brain-derived neurotrophic factor; DRD3: dopamine receptor D_3_; TRPC3: short transient receptor potential channel 3; DISC1: disrupted in schizophrenia 1; TDP-43: TAR DNA-binding protein 43.

A potential HERV impact on neurons was studied in mice using experimental overexpression of the HERV-K ENV protein, mimicking its expression in cortical and spinal neurons of ALS patients. These transgenic mice showed severe signs of neurodegeneration with progressive motor dysfunction, motor cortex volume loss, decreased synaptic activity, and spine abnormalities (Li et al., 2015). Such a phenotype implies that either endogenous damage pathways are activated or ENV protein leakage results in surface receptor activation, leading to autocrine or paracrine cell activation. CRISPR/Cas9 technology was recently used to disrupt the HERV-K ENV gene in human prostate cancer cells. By depleting ENV transcripts and proteins, this modification led to the downregulation of the above-mentioned important regulator TDP-43 (see **Figure 1**; Ibba et al., 2018). Given the formation of neurotoxic TDP-43 deposits in ALS neurons and TDP-43’s implication in HERV-K activation (Douville and Nath, 2017), this study provides yet more evidence for a role of ENV proteins in neurodegeneration. This view might, however, be challenged by the observation that HERV-K ENV overexpression in neuronal cells increased their viability and prevented neurotoxicity mediated by the HIV-1 Vpr protein (Bhat et al., 2014). This study was based on the fact that HERV-K and TDP-43 constitute an important neuropathological overlap between ALS and HIV encephalitis but might not be representative for MS- or ALS-related degeneration processes. To what degree inactivation of HERV-K might also be achieved *via* epigenetic modulators such as TRIM28 remains to be shown. In neural progenitor cells, TRIM28 acts a corepressor mediating transcriptional silencing. Its deletion resulted in induction of two groups of endogenous retroviruses IAP1 and MMERVK10C (Fasching et al., 2015). Finally, in neuroblastoma cells, HERV-W ENV overexpression was reported to activate the TRPC3 channel to regulate calcium influx and to depress the SCZ relevant DISC1 protein (Chen et al., 2019). Whether this observation truly reflects cellular expression and consequences in neuropsychiatric disorders including non-transformed neuronal cells and whether it can specifically be attributed to the envelope of HERV-W remain to be studied in future.

## Glial Cells

Expression of HERV-W ENV has mainly been observed in myeloid cells, i.e., monocytes/macrophages and microglia in MS patient tissues, while there is scarce evidence pointing to ENV expression by astrocytes (Perron et al., 2005; Kremer et al., 2013; Van Horssen et al., 2016). As of now, it is still unclear whether there is direct astroglial expression or if astroglia only bind or internalize ENV protein. However, signs of astroglial expression have also been gathered upon activation by EBV (Mameli et al., 2012). Observations on the related syncytin-1 revealed induced astrocytic release of redox reactants, which are cytotoxic to oligodendrocytes (Antony et al., 2004), possibly acting *via* ASCT1 activation (see **Figure 1**; Antony et al., 2007). In this regard, however, it must be stated that this study did not distinguished between the pathological HERV-W and the physiological syncytin-1. Furthermore, syncytin-1 overexpression in human microglia and astroglia was reported to activate the inflammatory marker CRP *via* TLR3 signaling (Wang et al., 2018), notably a receptor that is known to bind double-stranded RNA but is prone to nucleic acid artifacts in transfection experiments. In addition to the above-mentioned observations in neuroblastoma cells, overexpression of HERV-W ENV in human glioma cells was reported to induce expression of SCZ-linked genes encoding brain-derived neurotrophic factor (BDNF) and dopamine receptor D3 (DRD3; Huang et al., 2011), whereas the endogenous retroviral insert hsERVPRODH was found to act as a tissue-specific enhancer for the proline dehydrogenase 1 (PRODH), a candidate gene for SCZ susceptibility (Suntsova et al., 2013).

In the context of MS, stimulation of rat oligodendroglial progenitor cells (OPCs) with HERV-W ENV protein was found to impair their differentiation and to interfere with axon myelination (Kremer et al., 2013; Göttle et al., 2019). This effect is based on TLR4 activation and the subsequent induction of nitrosative stress (see **Figure 1**). The HERV-W ENV-targeted therapeutic antibody GNbAC1 was initially developed to neutralize ENV-dependent activation of immune cells, yet was also revealed to be active in rescuing oligodendroglial differentiation (Kremer et al., 2015) as well as myelination *in vitro* (Göttle et al., 2019). Of note, in relapsing MS patients, a phase 2b clinical trial using GNbAC1 has been conducted (CHANGE-MS, NCT02782858). It therefore remains to be shown to what degree clinical results reflect these preclinical findings and whether MS patients show beneficial effects on remyelination or attenuated neurodegeneration. Moreover, interfering with TLR4 surface exposition by blocking of the vascular ATPase was also found to neutralize the ENV-dependent effect on OPC differentiation and axonal myelination (Göttle et al., 2019). With regard to peripheral nervous system inflammatory damage, HERV-W ENV protein can also be detected in Schwann cells of CIDP patients. Cultured human Schwann cells exposed to or transfected with an ENV expression vector showed increased IL-6 and decreased CXCL10 transcript levels (Faucard et al., 2016), hence showing signs of altered immunocompetence in the peripheral nerve (Tzekova et al., 2014). Likewise, this activation could be neutralized *via* the GNbAC1 antibody (Faucard et al., 2016). To what degree HERV-W ENV protein-mediated activation also modulates the capacity of Schwann cells to de- and redifferentiate and whether it could therefore affect PNS repair remains to be shown.

Although microglial cells are currently viewed as a relevant source of HERV-W ENV protein in the diseased CNS, additional autocrine/paracrine effects on these myeloid cells cannot be excluded and warrant further investigations. In this regard, it is worth mentioning that nitric oxide (NO) production and cellular migration were found to be affected in response to stimulation of rat microglia with a recombinant ENV protein (Xiao et al., 2017).

Finally, a role of HERV-W ENV in diminishing myelin repair is also important in light of the reproduced documentation on its expression in MS but also considering recent findings implying an implication in molecular mimicry. In this regard, several groups provided evidence of similarities between HERV-W ENV and myelin oligodendrocyte glycoprotein (MOG). This molecular mimicry may be an underlying mechanism leading to or fueling autoimmunity (Do Olival et al., 2013; Ramasamy et al., 2017; De Luca et al., 2019). To what degree such molecular similarities also disturb successful maturation of resident OPCs required for myelin repair needs to be investigated in future.

## Conclusion

We here present collected evidence that endogenous retroviral elements acting either as viral particles or *via* their proteins influence neural cells in the context of degenerative CNS diseases. Once thought to be primarily involved in cell transformation (Grabski et al., 2019) and inflammation (Perron and Lang, 2010), emerging data suggests a direct role of these elements in glial and neuronal injury, which in fact goes beyond previous descriptions on the activity of a gliotoxin (Menard et al., 1998). In light of additional observations on the role of ERVs in regulating stem cell potential and fate acquisition (Gautam et al., 2017), the findings describing impacts on committed or mature cells of the CNS are probably not too surprising but warrant future investigations, even more so since neural stem cells are also involved in brain pathology and regeneration. Moreover, the currently still unmet clinical need to effectively treat neurodegeneration necessitates novel therapeutic approaches. Whether similar mechanisms also apply to activation of transposable elements implicated in, for example, chronic fatigue syndrome (CFS; Almenar-Perez et al., 2019) and to what degree currently used neutralizing antibodies can be exploited in order to prevent neural cell activation and/or neurodegeneration needs to be elucidated in the future. In this regard, it remains to be shown whether HERV-employed signaling pathways and epigenetic silencing mechanisms can be used for biomedical translation.

## Author Contributions

JG, DK, and PK searched the literature, interpreted manuscripts, and decided on the content of this review. JG and PK wrote the article. DK provided feedback on the manuscript draft and helped to complete the final manuscript. JG drew the figure.

## Funding

Research on myelin repair and HERVs in the laboratory of PK was mainly supported by the French societies ARSEP (Fondation pour l’Aide à la Recherche sur la Sclérose en Plaques) and AFM (Association Française Contre les Myopathies) as well as in part by Geneuro. JG is a student of the iBrain graduate school and PK and JG are supported by the Stifterverband/Novartisstiftung. DK was funded by the Deutsche Forschungsgemeinschaft (DFG) while carrying research on HERVs at Cleveland Clinic. The MS Center at the Department of Neurology is supported in part by the Walter and Ilse Rose Foundation and the James and Elisabeth Cloppenburg, Peek, and Cloppenburg Düsseldorf Stiftung.

## Conflict of Interest Statement

D.K. received compensation for speaking from Grifols SA. P.K. performed consultancy work for GeNeuro and received compensation for speaking from Sanofi Genzyme.

The remaining author declares that the research was conducted in the absence of any commercial or financial relationships that could be construed as a potential conflict of interest.

## References

[B1] Almenar-Perez E. Ovejero T. Sanchez-Fito T. Espejo J. A. Nathanson L. Oltra E. (2019). Epigenetic components of myalgic encephalomyelitis/chronic fatigue syndrome uncover potential transposable element activation. Clin. Ther. 41, 675–698. 10.1016/j.clinthera.2019.02.012 30910331

[B2] Antony J. M. Ellestad K. K. Hammond R. Imaizumi K. Mallet F. Warren K. G. (2007). The human endogenous retrovirus envelope glycoprotein, syncytin-1, regulates neuroinflammation and its receptor expression in multiple sclerosis: a role for endoplasmic reticulum chaperones in astrocytes. J. Immunol. 179, 1210–1224. 10.4049/jimmunol.179.2.1210 17617614

[B3] Antony J. M. Van Marle G. Opii W. Butterfield D. A. Mallet F. Yong V. W. (2004). Human endogenous retrovirus glycoprotein-mediated induction of redox reactants causes oligodendrocyte death and demyelination. Nat. Neurosci. 7, 1088–1095. 10.1038/nn1319 15452578

[B4] Arancio W. (2019). Progerin expression induces a significant downregulation of transcription from human repetitive sequences in iPSC-derived dopaminergic neurons. Geroscience 41, 39–49. 10.1007/s11357-018-00050-2 30623286 PMC6423266

[B5] Archer T. (2010). Neurodegeneration in schizophrenia. Expert Rev. Neurother. 10, 1131–1141. 10.1586/ern.09.152 20586693

[B6] Belshaw R. Pereira V. Katzourakis A. Talbot G. Paces J. Burt A. (2004). Long-term reinfection of the human genome by endogenous retroviruses. Proc. Natl. Acad. Sci. U.S.A. 101, 4894–4899. 10.1073/pnas.0307800101 15044706 PMC387345

[B7] Bhat R. K. Rudnick W. Antony J. M. Maingat F. Ellestad K. K. Wheatley B. M. (2014). Human endogenous retrovirus-K(II) envelope induction protects neurons during HIV/AIDS. PLoS One 9, e97984. 10.1371/journal.pone.0097984 24988390 PMC4079299

[B8] Bowen L. N. Tyagi R. Li W. Alfahad T. Smith B. Wright M. (2016). HIV-associated motor neuron disease: HERV-K activation and response to antiretroviral therapy. Neurology 87, 1756–1762. 10.1212/WNL.0000000000003258 27664983 PMC5089528

[B9] Brudek T. Luhdorf P. Christensen T. Hansen H. J. Moller-Larsen A. (2007). Activation of endogenous retrovirus reverse transcriptase in multiple sclerosis patient lymphocytes by inactivated HSV-1, HHV-6 and VZV. J. Neuroimmunol. 187, 147–155. 10.1016/j.jneuroim.2007.04.003 17493688

[B10] Brutting C. Narasimhan H. Hoffmann F. Kornhuber M. E. Staege M. S. Emmer A. (2018). Investigation of endogenous retrovirus sequences in the neighborhood of genes up-regulated in a neuroblastoma model after treatment with hypoxia-mimetic cobalt chloride. Front. Microbiol. 9, 287. 10.3389/fmicb.2018.00287 29515560 PMC5826361

[B11] Charvet B. Reynaud J. M. Gourru-Lesimple G. Perron H. Marche P. N. Horvat B. (2018). Induction of proinflammatory multiple sclerosis-associated retrovirus envelope protein by human herpesvirus-6A and CD46 receptor engagement. Front. Immunol. 9, 2803. 10.3389/fimmu.2018.02803 30574140 PMC6291489

[B12] Chen Y. Yan Q. Zhou P. Li S. Zhu F. (2019). HERV-W env regulates calcium influx *via* activating TRPC3 channel together with depressing DISC1 in human neuroblastoma cells. J. Neurovirol. 25, 101–113. 10.1007/s13365-018-0692-7 30397826

[B13] De Luca V. Martins Higa A. Malta Romano C. Pimenta Mambrini G. Peroni L. A. Trivinho-Strixino F. (2019). Cross-reactivity between myelin oligodendrocyte glycoprotein and human endogenous retrovirus W protein: nanotechnological evidence for the potential trigger of multiple sclerosis. Micron 120, 66–73. 10.1016/j.micron.2019.02.005 30802755

[B14] Derfuss T. Linington C. Hohlfeld R. Meinl E. (2010). Axo-glial antigens as targets in multiple sclerosis: implications for axonal and grey matter injury. J. Mol. Med. (Berl.) 88, 753–761. 10.1007/s00109-010-0632-3 20445955

[B15] Deslauriers A. M. Afkhami-Goli A. Paul A. M. Bhat R. K. Acharjee S. Ellestad K. K. (2011). Neuroinflammation and endoplasmic reticulum stress are coregulated by crocin to prevent demyelination and neurodegeneration. J. Immunol. 187, 4788–4799. 10.4049/jimmunol.1004111 21964030

[B16] Do Olival G. S. Faria T. S. Nali L. H. De Oliveira A. C. Casseb J. Vidal J. E. (2013). Genomic analysis of ERVWE2 locus in patients with multiple sclerosis: absence of genetic association but potential role of human endogenous retrovirus type W elements in molecular mimicry with myelin antigen. Front. Microbiol. 4, 172. 10.3389/fmicb.2013.00172 23805135 PMC3693062

[B17] Dolei A. (2018). The aliens inside us: HERV-W endogenous retroviruses and multiple sclerosis. Mult. Scler. 24, 42–47. 10.1177/1352458517737370 29307292

[B18] Dolei A. Perron H. (2009). The multiple sclerosis-associated retrovirus and its HERV-W endogenous family: a biological interface between virology, genetics, and immunology in human physiology and disease. J. Neurovirol. 15, 4–13. 10.1080/13550280802448451 19039700

[B19] Douville R. N. Nath A. (2017). Human endogenous retrovirus-K and TDP-43 expression bridges ALS and HIV neuropathology. Front. Microbiol. 8, 1986. 10.3389/fmicb.2017.01986 29075249 PMC5641584

[B20] Douville R. Liu J. Rothstein J. Nath A. (2011). Identification of active loci of a human endogenous retrovirus in neurons of patients with amyotrophic lateral sclerosis. Ann. Neurol. 69, 141–151. 10.1002/ana.22149 21280084 PMC3052883

[B21] Fasching L. Kapopoulou A. Sachdeva R. Petri R. Jonsson M. E. Manne C. (2015). TRIM28 represses transcription of endogenous retroviruses in neural progenitor cells. Cell Rep. 10, 20–28. 10.1016/j.celrep.2014.12.004 25543143 PMC4434221

[B22] Faucard R. Madeira A. Gehin N. Authier F. J. Panaite P. A. Lesage C. (2016). Human endogenous retrovirus and neuroinflammation in chronic inflammatory demyelinating polyradiculoneuropathy. EBioMedicine 6, 190–198. 10.1016/j.ebiom.2016.03.001 27211560 PMC4856744

[B23] Feschotte C. Gilbert C. (2012). Endogenous viruses: insights into viral evolution and impact on host biology. Nat. Rev. Genet. 13, 283–296. 10.1038/nrg3199 22421730

[B24] Frank O. Giehl M. Zheng C. Hehlmann R. Leib-Mosch C. Seifarth W. (2005). Human endogenous retrovirus expression profiles in samples from brains of patients with schizophrenia and bipolar disorders. J. Virol. 79, 10890–10901. 10.1128/JVI.79.17.10890-10901.2005 16103141 PMC1193590

[B25] Garson J. A. Tuke P. W. Giraud P. Paranhos-Baccala G. Perron H. (1998). Detection of virion-associated MSRV-RNA in serum of patients with multiple sclerosis. Lancet 351, 33. 10.1016/S0140-6736(98)24001-3 9433428

[B26] Garson J. A. Usher L. Al-Chalabi A. Huggett J. Day E. F. Mccormick A. L. (2019). Quantitative analysis of human endogenous retrovirus-K transcripts in postmortem premotor cortex fails to confirm elevated expression of HERV-K RNA in amyotrophic lateral sclerosis. Acta Neuropathol. Commun. 7, 45. 10.1186/s40478-019-0698-2 30885274 PMC6421708

[B27] Gautam P. Yu T. Loh Y. H. (2017). Regulation of ERVs in pluripotent stem cells and reprogramming. Curr. Opin. Genet. Dev. 46, 194–201. 10.1016/j.gde.2017.07.012 28866476

[B28] Göttle P. Förster M. Gruchot J. Kremer D. Hartung H. P. Perron H. (2019). Rescuing the negative impact of human endogenous retrovirus envelope protein on oligodendroglial differentiation and myelination. Glia 67, 160–170. 10.1002/glia.23535 30430656

[B29] Grabski D. F. Hu Y. Sharma M. Rasmussen S. K. (2019). Close to the bedside: a systematic review of endogenous retroviruses and their impact in oncology. J. Surg. Res. 240, 145–155. 10.1016/j.jss.2019.02.009 30933828 PMC9306217

[B30] Grandi N. Tramontano E. (2018). Human endogenous retroviruses are ancient acquired elements still shaping innate immune responses. Front. Immunol. 9, 2039. 10.3389/fimmu.2018.02039 30250470 PMC6139349

[B31] Huang W. Li S. Hu Y. Yu H. Luo F. Zhang Q. (2011). Implication of the env gene of the human endogenous retrovirus W family in the expression of BDNF and DRD3 and development of recent-onset schizophrenia. Schizophr. Bull. 37, 988–1000. 10.1093/schbul/sbp166 20100784 PMC3160218

[B32] Hurst T. P. Magiorkinis G. (2017). Epigenetic control of human endogenous retrovirus expression: focus on regulation of long-terminal repeats (LTRs). Viruses 9, 130. 10.3390/v9060130 PMC5490807 28561791

[B33] Ibba G. Piu C. Uleri E. Serra C. Dolei A. (2018). Disruption by SaCas9 endonuclease of HERV-Kenv, a retroviral gene with oncogenic and neuropathogenic potential, inhibits molecules involved in cancer and amyotrophic lateral sclerosis. Viruses 10, 412. 10.3390/v10080412 PMC6115762 30087231

[B34] Karlsson H. Bachmann S. Schroder J. Mcarthur J. Torrey E. F. Yolken R. H. (2001). Retroviral RNA identified in the cerebrospinal fluids and brains of individuals with schizophrenia. Proc. Natl. Acad. Sci. U.S.A. 98, 4634–4639. 10.1073/pnas.061021998 11296294 PMC31886

[B35] Kremer D. Förster M. Schichel T. Göttle P. Hartung H. P. Perron H. (2015). The neutralizing antibody GNbAC1 abrogates HERV-W envelope protein-mediated oligodendroglial maturation blockade. Mult. Scler. 21, 1200–1203. 10.1177/1352458514560926 25480862

[B36] Kremer D. Schichel T. Förster M. Tzekova N. Bernard C. Van Der Valk P. (2013). Human endogenous retrovirus type W envelope protein inhibits oligodendroglial precursor cell differentiation. Ann. Neurol. 74, 721–732. 10.1002/ana.23970 23836485

[B37] Kubben N. Zhang W. Wang L. Voss T. C. Yang J. Qu J. (2016). Repression of the antioxidant NRF2 pathway in premature aging. Cell 165, 1361–1374. 10.1016/j.cell.2016.05.017 27259148 PMC4893198

[B38] Küry P. Nath A. Creange A. Dolei A. Marche P. Gold J. (2018). Human endogenous retroviruses in neurological diseases. Trends Mol. Med. 24, 379–394. 10.1016/j.molmed.2018.02.007 29551251 PMC7185488

[B39] Li W. Lee M. H. Henderson L. Tyagi R. Bachani M. Steiner J. (2015). Human endogenous retrovirus-K contributes to motor neuron disease. Sci. Transl. Med. 7, 307ra153. 10.1126/scitranslmed.aac8201 PMC6344353 26424568

[B40] Linker R. A. Lee D. H. Ryan S. Van Dam A. M. Conrad R. Bista P. (2011). Fumaric acid esters exert neuroprotective effects in neuroinflammation *via* activation of the Nrf2 antioxidant pathway. Brain 134, 678–692. 10.1093/brain/awq386 21354971

[B41] Liu C. Liu L. Wang X. Liu Y. Wang M. Zhu F. (2017). HBV X protein induces overexpression of HERV-W env through NF-kappaB in HepG2 cells. Virus Genes 53, 797–806. 10.1007/s11262-017-1479-2 28639221

[B42] Liu C. L. Chen Y. T. Li S. Yu H. L. Zeng J. Wang X. L. (2013). Activation of elements in HERV-W family by caffeine and aspirin. Virus Genes 47, 219–227. 10.1007/s11262-013-0939-6 23813246

[B43] Macgowan D. J. Scelsa S. N. Imperato T. E. Liu K. N. Baron P. Polsky B. (2007). A controlled study of reverse transcriptase in serum and CSF of HIV-negative patients with ALS. Neurology 68, 1944–1946. 10.1212/01.wnl.0000263188.77797.99 17536052

[B44] Mackenzie I. R. Rademakers R. (2008). The role of transactive response DNA-binding protein-43 in amyotrophic lateral sclerosis and frontotemporal dementia. Curr. Opin. Neurol. 21, 693–700. 10.1097/WCO.0b013e3283168d1d 18989115 PMC2869081

[B45] Mak M. Samochowiec J. Frydecka D. Pelka-Wysiecka J. Szmida E. Karpinski P. (2019). First-episode schizophrenia is associated with a reduction of HERV-K methylation in peripheral blood. Psychiatry Res. 271, 459–463. 10.1016/j.psychres.2018.12.012 30537669

[B46] Mameli G. Astone V. Khalili K. Serra C. Sawaya B. E. Dolei A. (2007). Regulation of the syncytin-1 promoter in human astrocytes by multiple sclerosis-related cytokines. Virology 362, 120–130. 10.1016/j.virol.2006.12.019 17258784

[B47] Mameli G. Madeddu G. Mei A. Uleri E. Poddighe L. Delogu L. G. (2013). Activation of MSRV-type endogenous retroviruses during infectious mononucleosis and Epstein-Barr virus latency: the missing link with multiple sclerosis? PLoS One 8, e78474. 10.1371/journal.pone.0078474 24236019 PMC3827255

[B48] Mameli G. Poddighe L. Astone V. Delogu G. Arru G. Sotgiu S. (2009). Novel reliable real-time PCR for differential detection of MSRVenv and syncytin-1 in RNA and DNA from patients with multiple sclerosis. J. Virol. Methods 161, 98–106. 10.1016/j.jviromet.2009.05.024 19505508

[B49] Mameli G. Poddighe L. Mei A. Uleri E. Sotgiu S. Serra C. (2012). Expression and activation by Epstein Barr virus of human endogenous retroviruses-W in blood cells and astrocytes: inference for multiple sclerosis. PLoS One 7, e44991. 10.1371/journal.pone.0044991 23028727 PMC3459916

[B50] Manghera M. Ferguson-Parry J. Douville R. N. (2016a). TDP-43 regulates endogenous retrovirus-K viral protein accumulation. Neurobiol. Dis. 94, 226–236. 10.1016/j.nbd.2016.06.017 27370226

[B51] Manghera M. Ferguson-Parry J. Lin R. Douville R. N. (2016b). NF-kappaB and IRF1 induce endogenous retrovirus K expression *via* interferon-stimulated response elements in its 5’ long terminal repeat. J. Virol. 90, 9338–9349. 10.1128/JVI.01503-16 27512062 PMC5044829

[B52] Manghera M. Ferguson J. Douville R. (2015). ERVK polyprotein processing and reverse transcriptase expression in human cell line models of neurological disease. Viruses 7, 320–332. 10.3390/v7010320 25609305 PMC4306841

[B53] Mathis S. Couratier P. Julian A. Vallat J. M. Corcia P. Le Masson G. (2017). Management and therapeutic perspectives in amyotrophic lateral sclerosis. Expert Rev. Neurother. 17, 263–276. 10.1080/14737175.2016.1227705 27644548

[B54] Mccormick A. L. Brown R. H. Cudkowicz M. E. Al-Chalabi A. Garson J. A. (2008). Quantification of reverse transcriptase in ALS and elimination of a novel retroviral candidate. Neurology 70, 278–283. 10.1212/01.wnl.0000297552.13219.b4 18209202

[B55] Menard A. Amouri R. Dobransky T. Charriaut-Marlangue C. Pierig R. Cifuentes-Diaz C. (1998). A gliotoxic factor and multiple sclerosis. J. Neurol. Sci. 154, 209–221. 10.1016/S0022-510X(97)00231-1 9562313

[B56] Meyer T. J. Rosenkrantz J. L. Carbone L. Chavez S. L. (2017). Endogenous retroviruses: with us and against us. Front. Chem. 5, 23. 10.3389/fchem.2017.00023 28439515 PMC5384584

[B57] Mi S. Lee X. Li X. P. Veldman G. M. Finnerty H. Racie L. (2000). Syncytin is a captive retroviral envelope protein involved in human placental morphogenesis. Nature 403, 785–789. 10.1038/35001608 10693809

[B58] Perron H. Lang A. (2010). The human endogenous retrovirus link between genes and environment in multiple sclerosis and in multifactorial diseases associating neuroinflammation. Clin. Rev. Allergy Immunol. 39, 51–61. 10.1007/s12016-009-8170-x 19697163

[B59] Perron H. Dougier-Reynaud H. L. Lomparski C. Popa I. Firouzi R. Bertrand J. B. (2013). Human endogenous retrovirus protein activates innate immunity and promotes experimental allergic encephalomyelitis in mice. PLoS One 8, e80128. 10.1371/journal.pone.0080128 24324591 PMC3855614

[B60] Perron H. Geny C. Laurent A. Mouriquand C. Pellat J. Perret J. (1989). Leptomeningeal cell-line from multiple-sclerosis with reverse-transcriptase activity and viral particles. Res. Virol. 140, 551–561. 10.1016/S0923-2516(89)80141-4 2482522

[B61] Perron H. Germi R. Bernard C. Garcia-Montojo M. Deluen C. Farinelli L. (2012). Human endogenous retrovirus type W envelope expression in blood and brain cells provides new insights into multiple sclerosis disease. Mult. Scler. 18, 1721–1736. 10.1177/1352458512441381 22457345 PMC3573672

[B62] Perron H. Jouvin-Marche E. Michel M. Ounanian-Paraz A. Camelo S. Dumon A. (2001). Multiple sclerosis retrovirus particles and recombinant envelope trigger an abnormal immune response *in vitro*, by inducing polyclonal Vbeta16 T-lymphocyte activation. Virology 287, 321–332. 10.1006/viro.2001.1045 11531410

[B63] Perron H. Lazarini F. Ruprecht K. Pechoux-Longin C. Seilhean D. Sazdovitch V. (2005). Human endogenous retrovirus (HERV)-W ENV and GAG proteins: physiological expression in human brain and pathophysiological modulation in multiple sclerosis lesions. J. Neurovirol. 11, 23–33. 10.1080/13550280590901741 15804956

[B64] Perron H. Mekaoui L. Bernard C. Veas F. Stefas I. Leboyer M. (2008). Endogenous retrovirus type W GAG and envelope protein antigenemia in serum of schizophrenic patients. Biol. Psychiatry 64, 1019–1023. 10.1016/j.biopsych.2008.06.028 18760403

[B65] Perron H. Suh M. Lalande B. Gratacap B. Laurent A. Stoebner P. (1993). Herpes simplex virus ICP0 and ICP4 immediate early proteins strongly enhance expression of a retrovirus harboured by a leptomeningeal cell line from a patient with multiple sclerosis. J. Gen. Virol. 74 (Pt 1), 65–72. 10.1099/0022-1317-74-1-65 7678635

[B66] Ramasamy R. Joseph B. Whittall T. (2017). Potential molecular mimicry between the human endogenous retrovirus W family envelope proteins and myelin proteins in multiple sclerosis. Immunol. Lett. 183, 79–85. 10.1016/j.imlet.2017.02.003 28189601

[B67] Reich D. S. Lucchinetti C. F. Calabresi P. A. (2018). Multiple sclerosis. N. Engl. J. Med. 378, 169–180. 10.1056/NEJMra1401483 29320652 PMC6942519

[B68] Rolland A. Jouvin-Marche E. Saresella M. Ferrante P. Cavaretta R. Creange A. (2005). Correlation between disease severity and *in vitro* cytokine production mediated by MSRV (multiple sclerosis associated retroviral element) envelope protein in patients with multiple sclerosis. J. Neuroimmunol. 160, 195–203. 10.1016/j.jneuroim.2004.10.019 15710473

[B69] Ruprecht K. Obojes K. Wengel V. Gronen F. Kim K. S. Perron H. (2006). Regulation of human endogenous retrovirus W protein expression by herpes simplex virus type 1: implications for multiple sclerosis. J. Neurovirol. 12, 65–71. 10.1080/13550280600614973 16595376

[B70] Suntsova M. Gogvadze E. V. Salozhin S. Gaifullin N. Eroshkin F. Dmitriev S. E. (2013). Human-specific endogenous retroviral insert serves as an enhancer for the schizophrenia-linked gene PRODH. Proc. Natl. Acad. Sci. U.S.A. 110, 19472–19477. 10.1073/pnas.1318172110 24218577 PMC3845128

[B71] Sutkowski N. Conrad B. Thorley-Lawson D. A. Huber B. T. (2001). Epstein-Barr virus transactivates the human endogenous retrovirus HERV-K18 that encodes a superantigen. Immunity 15, 579–589. 10.1016/S1074-7613(01)00210-2 11672540

[B72] Trapp B. D. Peterson J. Ransohoff R. M. Rudick R. Mork S. Bo L. (1998). Axonal transection in the lesions of multiple sclerosis. N. Engl. J. Med. 338, 278–285. 10.1056/NEJM199801293380502 9445407

[B73] Tzekova N. Heinen A. Küry P. (2014). Molecules involved in the crosstalk between immune- and peripheral nerve Schwann cells. J. Clin. Immunol. 34 Suppl 1, S86–104. 10.1007/s10875-014-0015-6 24740512

[B74] Uleri E. Mei A. Mameli G. Poddighe L. Serra C. Dolei A. (2014). HIV Tat acts on endogenous retroviruses of the W family and this occurs *via* Toll-like receptor 4: inference for neuroAIDS. AIDS 28, 2659–2670. 10.1097/QAD.0000000000000477 25250834

[B75] Van Horssen J. Van Der Pol S. Nijland P. Amor S. Perron H. (2016). Human endogenous retrovirus W in brain lesions: rationale for targeted therapy in multiple sclerosis. Mult. Scler. Relat. Disord. 8, 11–18. 10.1016/j.msard.2016.04.006 27456869

[B76] Wang X. Liu Z. Wang P. Li S. Zeng J. Tu X. (2018). Syncytin-1, an endogenous retroviral protein, triggers the activation of CRP *via* TLR3 signal cascade in glial cells. Brain Behav. Immun. 67, 324–334. 10.1016/j.bbi.2017.09.009 28928004

[B77] Weis S. Llenos I. C. Sabunciyan S. Dulay J. R. Isler L. Yolken R. (2007). Reduced expression of human endogenous retrovirus (HERV)-W GAG protein in the cingulate gyrus and hippocampus in schizophrenia, bipolar disorder, and depression. J. Neural Transm. (Vienna) 114, 645–655. 10.1007/s00702-006-0599-y 17219017

[B78] Xiao R. Li S. Cao Q. Wang X. Yan Q. Tu X. (2017). Human endogenous retrovirus W env increases nitric oxide production and enhances the migration ability of microglia by regulating the expression of inducible nitric oxide synthase. Virol. Sin. 32, 216–225. 10.1007/s12250-017-3997-4 28656540 PMC6598877

[B79] Yolken R. H. Karlsson H. Yee F. Johnston-Wilson N. L. Torrey E. F. (2000). Endogenous retroviruses and schizophrenia. Brain Res. Rev. 31, 193–199. 10.1016/S0165-0173(99)00037-5 10719148

[B80] Yu H. Liu T. Zhao Z. Chen Y. Zeng J. Liu S. (2014). Mutations in 3’-long terminal repeat of HERV-W family in chromosome 7 upregulate syncytin-1 expression in urothelial cell carcinoma of the bladder through interacting with c-Myb. Oncogene 33, 3947–3958. 10.1038/onc.2013.366 24013223

